# Population control by means of organised hunting effort: Experiences from a voluntary goose hunting arrangement

**DOI:** 10.1007/s13280-021-01590-2

**Published:** 2021-06-29

**Authors:** Ingunn M. Tombre, Fredrik Fredriksen, Odd Jerpstad, Jan Eivind Østnes, Einar Eythórsson

**Affiliations:** 1 Department of Arctic Ecology, The Fram Centre, Norwegian Institute for Nature Research, Langnes, P.O. Box 6606, 9007 Tromsø, Norway; 2 NORD University, P.O. Box 2510, 7729 Steinkjer, Norway; 3 Forum for Nature and Outdoor Life in Trøndelag, Kjøpmannsgata 12, 7500 Stjørdal, Norway; 4 Brandsåsvegen 86, 7724 Steinkjer, Norway; 5 Faculty of Biosciences and Aquaculture, NORD University, P.O. Box 2510, 7729 Steinkjer, Norway; 6 High North Department, The Fram Centre, Norwegian Institute for Cultural Heritage Research, Langnes, P.O. Box 6606, 9007 Tromsø, Norway

**Keywords:** Adaptive framework, Geese, Local engagement, Management implementation, Optimal hunting arrangements, Recreational hunting

## Abstract

Implementing management objectives may be challenging when decisions are made at different scales than where they are supposed to be carried out. In this study we present a situation where local goose hunting arrangements respond to objectives in an international management plan for pink-footed geese (*Anser brachyrhynchus*) and a local wish to reduce goose numbers as means to reduce grazing damage on farmland. A unique ten-year dataset provides an evaluation of the efficiency of voluntary actions at a local scale for implementing a policy of population control of geese, and general lessons are drawn for collaboration and co-production of knowledge for adaptive management. The study demonstrates how both the hunters and geese adapt in a situation where increasing the harvest of geese is the main objective. Introducing hunting-free days and safe foraging areas significantly increased goose numbers in the study area, with a corresponding increase in hunting success in terms of number of harvested geese. The geese’s behavioural response to hunting also triggered the hunters to adapt accordingly by optimal timing and placement in the landscape. Based on the results of the present study we suggest a framework for local implementation of management actions. Bringing end-users on board, facilitates processes and strengthens the achievements, as they represent the actors where implementation occurs. Specifically, our findings demonstrate how optimal goose hunting can be practiced by the use of an adaptive framework with active stakeholder participation.

## Introduction

Local engagement is a significant contributor to successful implementation of management actions (Pagdee et al. 2006; Andrade and Rhodes 2012; Caro and Davenport 2015). In wildlife management, where conflicting interests have to be weighed against each other (Conover 2002; Decker et al. 2012), an adaptive process of learning and stakeholder involvement can facilitate this (Failing et al. 2004; Berkes 2009; Williams 2011). However, development and implementation of management actions usually occur at different scales, and management measures need to be adapted to local conditions (Lessard 1998; Decker et al. 2005; Cumming et al. 2012; Redpath et al. 2018). Cross-scale collaboration depends on information sharing, open communication and transparent processes to enhance the local engagement. Local participants must also be willing to participate in the management processes and be able to adapt. All these aspects are rarely fulfilled. There are few examples where such a framework is practiced successfully (but see e.g. Hahn et al. 2006; Tuvendal and Elmberg 2015).

The significant increase in most of the Western-Palearctic wild goose populations is a wildlife management challenge. Initiatives for management should aim at balancing sustainable populations while reducing conflicts with human interests and other biodiversity (Fox and Madsen 2017). As conflicts arise due to increasing goose numbers interfering with agricultural interests, airport safety and biodiversity objectives (Fox and Madsen 2017; Madsen et al. 2017; Powolny et al. 2018; Jensen et al. 2018), measures for population control have become increasingly relevant and are now implemented for several goose populations (Reed and Calvert 2007; Leafloor et al. 2012; Lefebvre et al. 2017; Madsen et al. 2017). As a part of an international management plan for the Svalbard-breeding population of pink-footed geese (*Anser brachyrhynchus*) adopted under the Agreement on the Conservation of African-Eurasian Migratory Waterbirds, a target of 60 000 individuals in spring has been agreed among the range states hosting the population (Madsen and Williams 2012). By stabilising the population around this level, the aim is to reduce conflicts with agriculture and limit tundra degradation on the breeding grounds, challenges that have grown with increasing goose numbers (Madsen and Williams 2012). Pink-footed geese are a source for conflicts with the agricultural interests in Norway as they forage on vulnerable crops on stopover sites during spring migration (Tombre et al. 2013b; Madsen et al. 2014; Eythórsson et al. 2017; Simonsen et al. 2016, 2017) and reduce harvest yields (Bjerke et al. 2014; Olsen et al. 2017). There are also signs of tundra degradation on the breeding grounds on Svalbard (Speed et al. 2009; Pedersen et al. 2013a, b).

When the management plan for pink-footed geese was endorsed in 2012, the population counted more than 80 000 individual geese (Madsen et al. 2017). As one objective in the plan is to allow recreational hunting that does not jeopardise the population, more efficient autumn hunting in Denmark and Norway was decided as a measure to regulate the population. Autumn hunting is an established wildlife management tool in these countries, while the species is protected in the southernmost wintering range states; the Netherlands and Belgium (Madsen et al. 2015, 2017). At present (2020), numbers are estimated to be around 75 000 individuals (Heldbjerg et al. 2019). Hence, the population size is still above the target, meaning that an increased harvest rate is an important management action. The typical means available to achieve this goal are rules and regulations set by national wildlife authorities, such as hunting quotas, bag limits, length of hunting season and type of hunting weapons and ammunition allowed. Landowners, who regulate access to hunting on private land, can also set local rules on hunting hours and allowed hunting practices. In some areas, voluntary landowners’ associations (LOAs) have implemented such local regulations. Mutual learning involving biological and cross-disciplinary research as well as experience-based knowledge, is essential for the development of a workable hunting regime, considering goose response to disturbance, landscape and property structure and hunters’ acceptance of a management role (Søreng et al. 2013, 2015; Holmgaard et al. 2018). The process also depends upon the creation of voluntary administrative solutions to monitor hunting practice and provide neighbourhood safety. Information sharing between managers, scientists and local actors are therefore crucial to build trust and sense of ownership of the process.

In this paper, we present a situation where local implementation of goose hunting arrangements responds to objectives in the international management plan for pink-footed geese by following an adaptive process of gaining experiences and learning. We present results from experimental applications of different hunting practices aiming at more efficient hunting of geese and less disturbance, carried out by a LOA in Egge, a neighbourhood in Steinkjer municipality in the northern part of Trøndelag County in central Norway. Trøndelag County is the main autumn stopover site for pink-footed geese and counts for more than 80% of the annual pink-footed goose harvest in Norway (Tombre et al. 2017). In this rural region, with a mixture of cereal fields (dominated by barley) and dairy farming, however, the farms are often fragmented in a way that is an obstacle for efficient goose hunting arrangements over larger areas, as disturbance from hunting at one farm easily scares the geese off the neighbouring properties.

The Egge LOA, consists of seven farmers and has kept a statistical record of several parameters for hunting practices in the area from 2010 to 2019. We have combined these records with other available sources (goose registrations and official statistics) in order to quantify and evaluate the effects of local hunting practices on bag statistics as well as the goose behavioural response to hunting, with corresponding effects on bag sizes. We expect that the number of harvested geese reflects the controlled hunting practice as seen in Jensen et al. (2016a, b). However, the total number of geese available for hunting may also influence this, and the number of geese staging in the area is therefore also evaluated with respect to the hunting bag. Number of pink-footed geese in Egge is also seen in light of the changes in the total population size. To evaluate the challenges and advantages in the development of local goose hunting arrangements, information from interviews and surveys among local farmland owners and hunters in the region is also included. Landowners are the key for optimal arrangements since the majority of the autumn-staging geese in Trøndelag forage on private land. Perspectives from the survey and interviews will therefore provide information about the realism of implementing local hunting arrangements. The goose hunters are also significant contributors for successful implementation, as they are the actors who must adapt to the existing hunting regimes. The Egge case started out with equal access for all hunters, resulting in high hunting intensities in 2008–2013 in terms of many hunters hunting for several consecutive days at several sites in the area. The LOA changed its access policy in 2014, towards limiting access to one coordinated hunting team and decided to test a model with lower hunting intensities with more hunting-free days. This was based on their previous experiences, as well as gained information from recent research (Jensen et al. 2016a, b, 2017), indicating that lower hunting intensity is instrumental for an increase in total number of geese harvested.

The study represents a unique dataset providing an evaluation of the efficiency of voluntary actions at a local level for implementing a policy of population control within a framework of an international management plan. Hence, from this documentation, we do not only gain information about optimal goose hunting arrangements but also draw general lessons for collaboration and co-production of knowledge for adaptive management.

## Materials and methods

### Study area and goose species

**Fig. 1:**
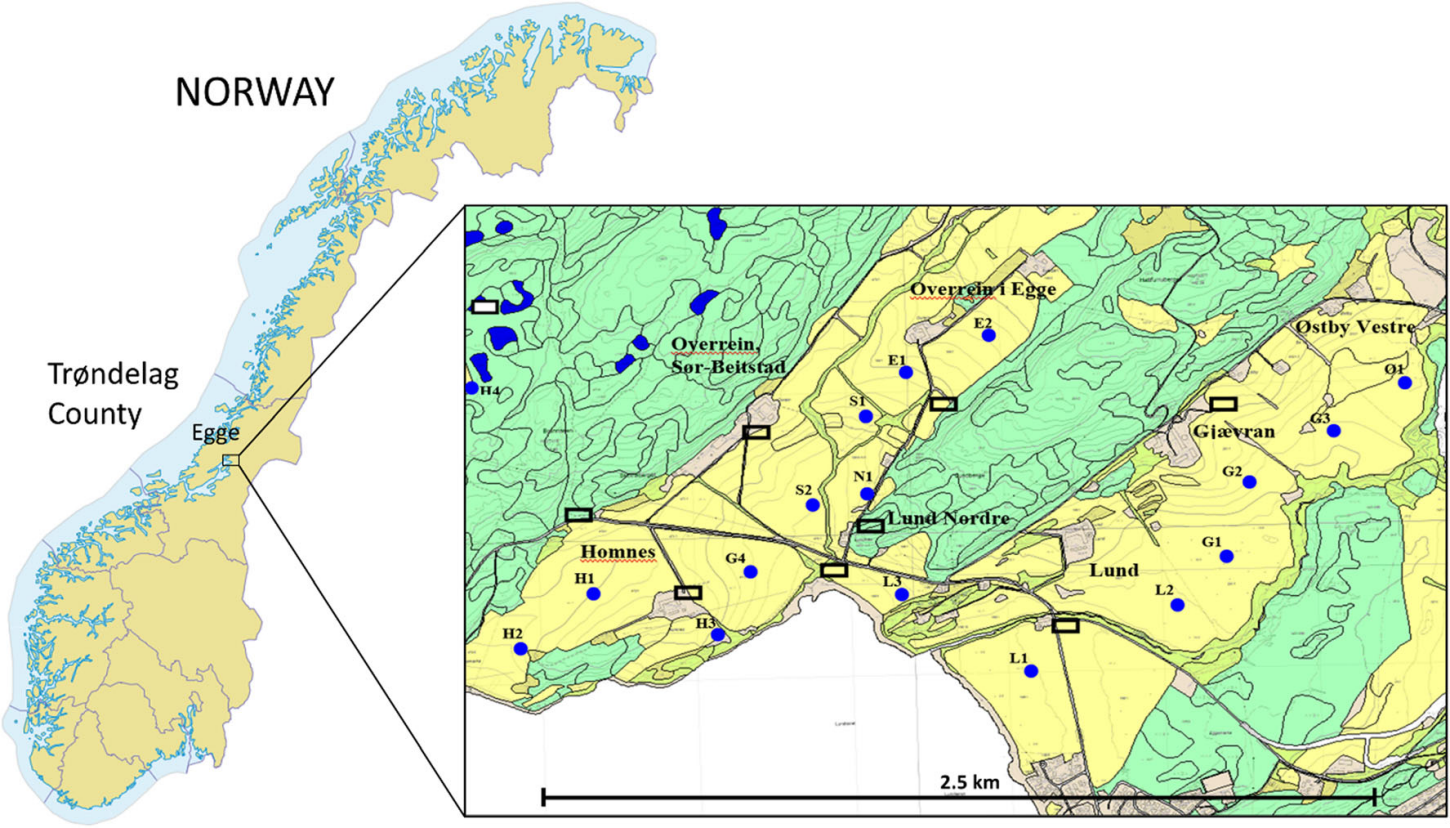
The study site Egge, in the county of Trøndelag in Central Norway. The inserted map is the map given to the goose hunters. Yellow areas are farmland area, primarily barley fields, where the geese forage at daytime and the hunting is practiced covering approximately 2 km^2^. Blue spots indicate the different hunting fields with separate IDs. Also shown, as black squares, are locations where the hunters can park their car further facilitating the hunting arrangement. The bay and the seashore, a bird protection area, are the roosting site for geese

The study was carried out in Trøndelag county in central Norway (Fig. 1). The region is a rich farmland area with cereals, pastures and root vegetables as the dominating crops. In the autumn, harvested fields provide food for autumn-staging geese in terms of spilt grain on stubble fields (Jensen et al. 2016b). Most of the hunting occurs on such fields, where hunters use shotguns from blinds shooting on individual geese entering the farmland fields in smaller flocks from their nightly roosting sites in the morning.

The farmland area at Egge, our core study area, ranges approximately 2 km inland from the shoreline and in total consists of around 2 km^2^ of cereal fields, primarily barley (Fig. 1). Two clusters of cereal fields are separated by a 300–500 m wide ridge and smaller roads, and are surrounded by dense settlements and roads to the east, forests to the north and west, and a fjord in the south, making the areas used by geese isolated and surveyable. Hence, it is relatively easy to keep track of the geese, and to register the numbers and their spatial distribution. Seven farmers participate in the goose hunting organisation at Egge, a cluster originally established for moose hunting but expanded in 2008 to also include hunting on geese.

In the winter season, this population of pink-footed geese stays in Belgium, the Netherlands and Denmark, whereas spring staging occurs in Denmark and Norway, breeding on the arctic archipelago Svalbard and autumn staging in Norway and Denmark. In recent years, an increasing number of pink-footed geese also migrate through Sweden and Finland (Heldbjerg et al. 2019). Pink-footed geese were registered for the first time in Trøndelag in the 1990s (Madsen et al. 1999). In autumn, pink-footed geese arrive in Mid-Norway and the Trøndelag region in mid-September, and flocks can be observed in the area until December although the majority of geese depart the region in late October (Jensen et al. 2016b).

Greylag geese (*Anser anser*) breed on mainland Norway and do not migrate to high-arctic breeding grounds on Svalbard as the pink-footed geese. However, they also stage in Trøndelag in the autumn, mainly from mid-August to October (Jensen et al. 2016a, b; Tombre et al. 2016). There is an open hunting season also for this species, and the number of harvested greylag geese from the Trøndelag region has increased (Statistics Norway, www.ssb.no/en).

### Goose hunting and goose hunting arrangements

Relevant background information regarding perspectives on goose management, goose hunting, and the engagement and motivation for local actions were collected from in-depth interviews and surveys from hunters, farmland owners and local managers in Trøndelag over the period 2008–2019. Some related results from farmland owners and local managers have previously been published in Norwegian technical reports (Tombre et al. 2009, 2011; Eythórsson and Tombre 2013; Søreng et al. 2013, 2015) whereas results from a hunter survey have been published in a peer-review journal (Holmgaard et al. 2018). Here, we focus on experiences in the development of local goose hunting practices and arrangements previously not published, and also include knowledge from in-depth interviews with a farmland owner and a hunter from another goose hunting arrangement in a different municipality in Trøndelag (the focus case in Jensen et al. 2016a, b, 2017). Pink-footed geese and greylag geese have been systematically monitored in the Trøndelag region over the period 2014–2019 (Tombre et al. 2017; Tombre and Gundersen, unpubl.), and during this fieldwork, information about goose hunting in general and specific hunting practices have also been gathered from several local farmland owners and hunters.

The data on hunting practices in Egge gives a complete overview of all the goose hunters, including spatial and temporal bag statistics on a daily basis. Over the years 2014–2019, goose hunting was organised introducing hunting-free days, and experiences with different hunting arrangements were collected and shared with the LOA. In these years, geese were searched for several times a day (see details for specific count data) to assess distribution on the farmland fields, and the field used in the afternoon was selected for hunting the next morning as geese tend to come back to the same field the following day. Hence, hunting took place after an estimation of total number of geese in the area and after an assessment of their field preferences. Some fields were better than others for hunting, due to a wider shooting angle and a more optimal topography in terms of shooting distances. In general, hunting was planned if the number of geese in the area exceeded approximately 500 individuals. This was based on previous experiences of less hunting success if there were fewer geese in the area. Depending on the time of season, however, hunting was also conducted when there were fewer geese, if it was unlikely that more geese would arrive.

### Harvest data

For Egge, the yearly numbers of hunting events, total number of geese harvested and number of geese harvested per hunting event was collected for a ten years period (2010–2019). As there was a change in hunting practice in 2014, we compared the two time periods 2010–2013 and 2014–2019.

At the municipality level, harvest data is available from Statistics Norway, and these figures are used to estimate potential changes in harvest from Egge in relation to the municipality level for Steinkjer.

### Goose counts

The goose registrations used in the study are from different sources. In 2014–2016, Egge was scouted for geese every day from the first observations in August until the geese leave the area in October. Once a day during this period, geese were counted using binoculars, telescope and a hand clicker while resting along the seashore at high tides within the period 17 August to 20 October. These registrations were the basis for the hunting plan, whereas registrations within 1 September and 10 October are used to calculate averages of geese in the area each year. In 2010, 2011, 2013 and 2017, numbers were based on data from an online data portal (www.artsobservasjoner.no), a species reporting system for voluntary observers. If there were two or more different registrations of geese (pink-footed geese or greylag geese) from the Egge area on the same day from different observers, the highest number was selected. This resulted in registrations from three to five different observers each year. As the study area is (I) limited in geographical range with a landscape topography well suited for bird observations and (II) the majority of observations were from the roosting site at the seashore, we anticipate that these figures provide representative numbers of geese in the area. In 2012, goose numbers were collected at a daily basis between 17 September and 5 October, and in 2018 and 2019, the counts were conducted between 1 September and 16 October with five and eight counts, respectively. The short count period in 2012 was a result of a designed project within the core stopover period for geese, and we assume that these days will be representative and comparable with the other years having the main registrations within the same period. Based on these goose observations, we select a core period between 1 September and 10 October, also being the core period for goose hunting, and calculate the average goose numbers for each year as a measure for goose presence in the study area. The number of geese present may also affect the number of geese harvested, and averages were hence compared between the two periods 2010–2013 and 2014–2019 as these periods represent two different hunting practices.

### Hunter behaviour and goose response to hunting

The hunter behaviour data collected was the total number of hunting days, the number of different hunters and the number of geese shot per hunting event. This information also gave an overview of number of fields occupied by hunters, the number of occupied fields with successful hunting, i.e. where at least one goose was harvested each hunting event. Efficiency of field-use equals the number of successful hunting events divided by the number of used fields.

For the years 2014–2016, the behavioural response of geese to the hunting activities was analysed based on the data from daily goose registrations, i.e. their numbers and distribution in the area. Distances between the localization of the hunting team and goose flocks were quantified by marking locations on a map and later measured to the nearest 5 m. The distances between fields used by geese from one day to the next were compared between days with hunting, and days without hunting.

### Statistical analyses

A set of linear regression analyses were conducted to quantify effects of the hunting practice over the years. By these analyses we can calculate the coefficients of determination (R-square) describing how much of the variance in the response variables is explained by the year effect. The various response variables are listed in a table showing the regression coefficients, parameter estimates and p-values. To evaluate the pink-footed goose numbers in the study area in relation to the total population size (data extracted from Heldbjerg et al. 2019), we also performed a regression analysis. This was not possible for greylag geese as there are no yearly size assessments of this population.

In cases with possible covariance between several variables, general linear models (GLM, Type III Sum of Squares) were carried out to test for combined effects. Comparing the before-and-after situation with 4 and 6 years in each category within a time period of ten years, gives a limited sample size. For each before- and after-period we have calculated averages of goose numbers, bag sizes in Egge, and the bag size percentage in Egge in relation to the municipality level, and where appropriate also conducted student *t-*test for comparisons. Analyses were carried out using the statistical software SAS 9.4.

## Results

### The development of goose hunting practices in Trøndelag

The interviews revealed that when autumn-staging pink-footed geese were registered for the first time in Trøndelag, goose hunting was not a part of the local hunting tradition. Motivated by the large flocks of spring-staging geese causing substantial crop damages in parts of the region, many farmers welcomed hunters to their farms. Hence, in the autumn hunting season, from 10 August to 23 December, hunters could access farmland fields by contacting individual farmers, who usually granted permission to hunt free of charge or against a symbolic payment. Many farmland owners reported that easy access to goose hunting attracted both experienced and unexperienced hunters, but the unregulated hunting practices soon provoked negative reactions. Some hunters did not bother asking for permission, and even drove into wet fields at night and damaged the farmland. Serious safety concerns were raised as some hunters also appeared to shoot in all directions on fields within shooting range to roads and houses.

A process towards regulation of goose hunting in Trøndelag through LOAs started in 2008 as an initiative from the regional wildlife authorities (The County Governor). At first, access limitations were not prioritized since the apparent challenge was to encourage more hunters to participate. In the case of Egge, the local goose hunting arrangement developed based on a pre-existing association organizing moose hunting.

From the survey among landowners, respondents who were engaged in LOA initiatives with regulated goose hunting claimed to have low expectations for potential income from hunting. They explained their engagement by the need to regulate the goose population to limit crop damages and by safety concerns. In the long run, however, a mismatch between low income and the amount of voluntary work needed for administration, monitoring and to facilitate for hunting made it challenging to maintain open card sale arrangements. Reduction of workload for LOA-members was thus a part of the motivation for restricting access by only allowing organized hunting teams and to delegate some of the monitoring tasks to team leaders.

### The goose hunting arrangement in Egge

Following the County Governor’s advice and responding to an increasing interest from hunters, landowners in Egge organised joint sale of hunting permits in the autumn 2008. From 2008–2013, the LOA practiced equal access, meaning that hunters asking for hunting permission were given access, provided that there were vacant hunting posts on the relevant day. At first, hunting was open any time of day, but from 2010, afternoon and evening hunt was forbidden based on the hunting reports showing that few geese were shot at this time of day. In addition, the hunting disturbance affected the goose abundance and corresponding hunting success the next morning. This was an advice also gained from research results in the region. The morning hunt was then decided to be between 06.00 AM and 12.00 PM on weekdays, and from 07.00 AM to 12.00 PM in the weekends. The hunting area followed the farmland landscape and was divided in different hunting fields with a limited number of hunting posts. Hunting fields were identified based on goose presence and where it was feasible to hunt in respect to safety and expected success. Attached to the hunters’ agreement was a map (Fig. 1) where these fields were indicated. Also the recommended, and preferred, car parking locations were shown as a challenge for landowners is often the cars parked at the farmyard. The seashore in Egge is a bird protection area, and hunters were also informed about the ban on all kinds of hunting and use of weapons in this area. Information signs in the field also illustrated the borders to the protected area where they were on land.

From 2014 access was limited to one hunting party of three to five hunters, in charge of all goose hunting in the area. The aim was to organise hunting in a way that maximised the number of harvested geese, an important objective for the farmers and in accordance with the current status of the international management plan. Several hunting-free days were introduced in order to reduce disturbance and thus prevent the goose flocks from spreading to other areas. On hunting days, the team established the equipment (ground blinds and decoys attracting the incoming goose flocks) on the stubble field two hours before daylight (between 03.00 and 05.00 AM depending on the date as this determines light conditions). As far as possible, the party consisted of the same experienced hunters, although there were some replacements during the study period. The leader of the hunting team was the same (in 2014–2019). Almost without exceptions, the geese came from the roosting area on the seashore to feed on surrounding fields in the morning, most often leading to several shooting opportunities as the different flocks approached the field. Following the Norwegian hunting law, a maximum of two shots per shotgun was used before reloading. The hunt ended when it seemed unlikely that new shooting opportunities would appear (usually after one hour of waiting), or at the latest at 12.00 PM.

A premise for hunting at Egge, throughout the whole study period, was that hunters reported their bag to the LOA, in addition to the mandatory reporting to Statistics Norway. Information from Statistics Norway is open access and harvest data can be downloaded at the county and municipality level. Hence, for the LOA level, landowners have to ask for reports from the hunters.

### Hunting practices and harvested geese in Egge

**Fig. 2:**
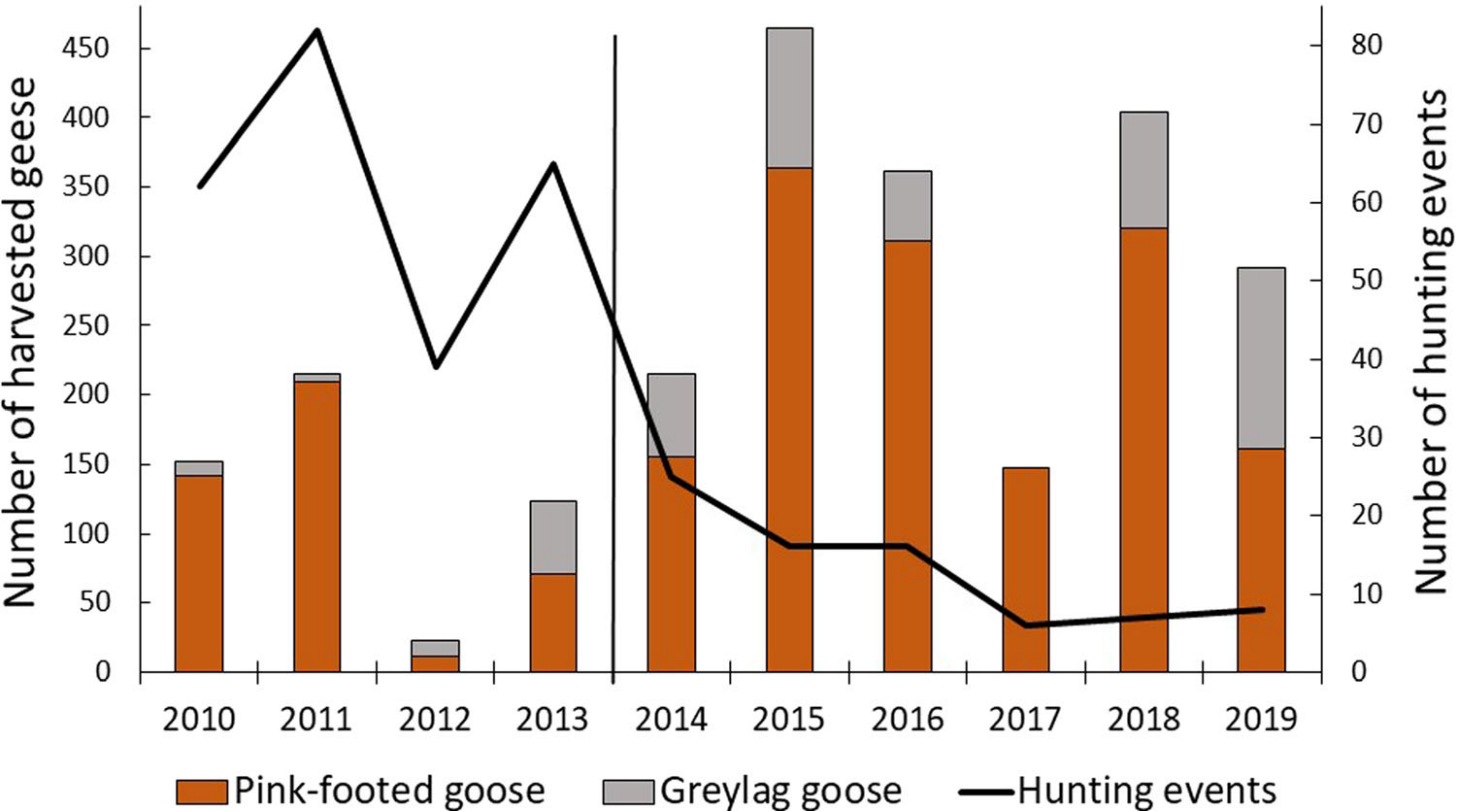
The number of pink-footed and greylag geese harvested each year from 2010 to 2019 in Egge, Trøndelag County, Norway. Also shown is the total number of hunting events (number of times a hunting team is out hunting) for the same years. The vertical line separates years of different hunting practices. From 2014 and onwards, only one hunting team was hunting, introducing hunting-free days between the hunts

**Fig. 3:**
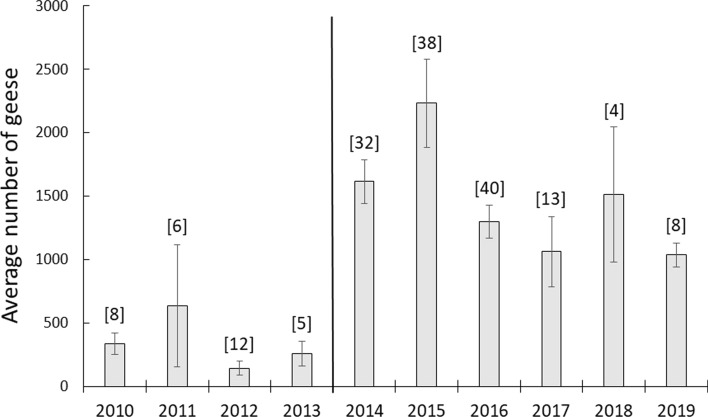
The average number of geese observed per year in Egge, Trøndelag County, Norway. Averages are based on both pink-footed geese and greylag geese. Numbers on top of each column are number of observation days within the period 1 September–10 October, the core hunting and goose period in the area. The vertical line separates years of different hunting practices. From 2014 and onwards, only one hunting team was hunting, introducing hunting-free days between the hunts

The numbers of geese harvested each year in Egge are shown in Fig. 2 and Table 1 for the years 2010–2019. The majority of the harvest is pink-footed geese, although the number of greylag geese has increased in the bag over the years. Comparing the two periods, before and after the change in hunting arrangement when more hunting free days were introduced, showed that significantly more geese were shot after this change (before: 128.5 ± 40.0, *n* = 4, after: 314.2 ± 48.8, *n* = 6, t =  − 2.70, *p* = 0.027). There was also a positive trend over the years, although the regression was not significant (Table 2). The number of hunting events, a measure of hunting pressure in terms of the number of times a hunting team is out hunting, decreased over the years (Table 2). Average number of hunting events was significantly less in the period when hunting was organised with hunting free days (before: 62.0 ± 8.8, *n* = 4, after: 13.0 ± 3.0, *n* = 6, *t* = 6.17, *p* = 0.0003). Very few geese staged in Egge in 2012 (Fig. 3), causing a drop in hunting events compared to the previous year (2011), a year having the highest number of hunting events during the study period (Fig. 2). Fewer geese were also harvested in 2017, compared to adjacent years. It was a year where, in spite of the geese observed (Fig. 3), fewer greylag geese and late arriving pink-footed geese that continued the southward migration earlier, gave fewer hunting opportunities. In total, however, more geese were harvested in 2017 during six hunting events than during the 65 events in 2013 (Fig. 2, Table 1).

**Table 1:** The number of hunting events, total number of harvested geese and the number of harvested geese per hunting event for each year 2010–2019, and averaged for two time periods (± SE) in Egge, in the county of Trøndelag, Norway. The 4 and 6 year periods in 2010–2013 and 2014–2019, respectively, represent two different hunting regimes where the latter also includes hunting-free days. The harvested geese are both pink-footed geese and greylag geese

Year	Number of hunting events	Total number of harvested geese	Number of harvested geese per hunting event
2010	62	152	2.5
2011	82	215	2.6
2012	39	23	0.6
2013	65	124	1.9
2014	25	215	8.6
2015	16	465	29.1
2016	16	362	22.6
2017	6	147	24.5
2018	7	404	57.7
2019	8	292	36.5
2010–2013	62.0 ± 8.8	128.5 ± 40.0	2.1 ± 0.5
2014–2019	13.0 ± 3.0	314.2 ± 48.8	24.2 ± 6.7

**Table 2:** Linear regression analyses revealing trends over a 10-year period (2010–2019) of different response variables in Egge, in the county of Trøndelag, Norway. Also shown is the relationship between the total population size of pink-footed geese (pinkfeet) and the average number of pinkfeets observed each year in the study area. Regression coefficients, parameter estimates and *p*-values are shown

Predictor variable	Response variable	R-square	Estimate	*p*
Year	# harvested geese	0.30	25.3	0.009
Year	# hunting events^a^	0.77	− 8.2	0.001
Pop.size pinkfeet	Average pinkfeet numbers	0.01	0.01	0.801
Year	# fields with hunters	0.77	− 8.1	0.001
Year	# events with success	0.67	− 2.5	0.004
Year	% efficiency of fields used	0.80	7.7	0.001
Year	# hunting days	0.76	− 12.6	0.001
Year	# hunters	0.65	− 3.8	0.005
Year	# geese shot per day	0.75	5.4	0.001

^a^The number of times a hunter is out hunting within each year

The increasing number of harvested geese after the hunting arrangement was organized with hunting-free days may, however, also be a result of more geese in the area. Figure 3 illustrates the sudden increase in goose numbers from 2014 and onwards matching the changes in hunting practice (2010–2013: 343.2 ± 105.1, *n* = 4, 2014–2019: 1459.8 ± 181.8, *n* = 6, *t* =  − 4.61, *p* = 0.002). In a model including both the year, hunting events and average goose numbers, the goose numbers are also the variable demonstrating a significant relationship when the other variable are controlled for (GLM, Type III SS, Year; *F*_2,9_ = 2.16, estimate = 25.6, *F* = 0.16, Average goose number; F_2, 9_ = 14.97, estimate = 6.2, *p* = 0.008, Hunting events; *F*_2,9_ = 2.13, estimate = 3.1, *p* = 0.195). More geese in the area may be a result of the changed hunting practice, as more hunting-free days provide more safe refuges for the geese. Average numbers of pink-footed geese were not, however, related to the total population size (Table 2) which has increased from 69 000 individuals in 2010 to 76 500 individuals in 2019. Hence, at least for pink-footed geese, the dominating species in Egge, the increase in numbers does not follow the increase of the population as a whole.

**Fig. 4:**
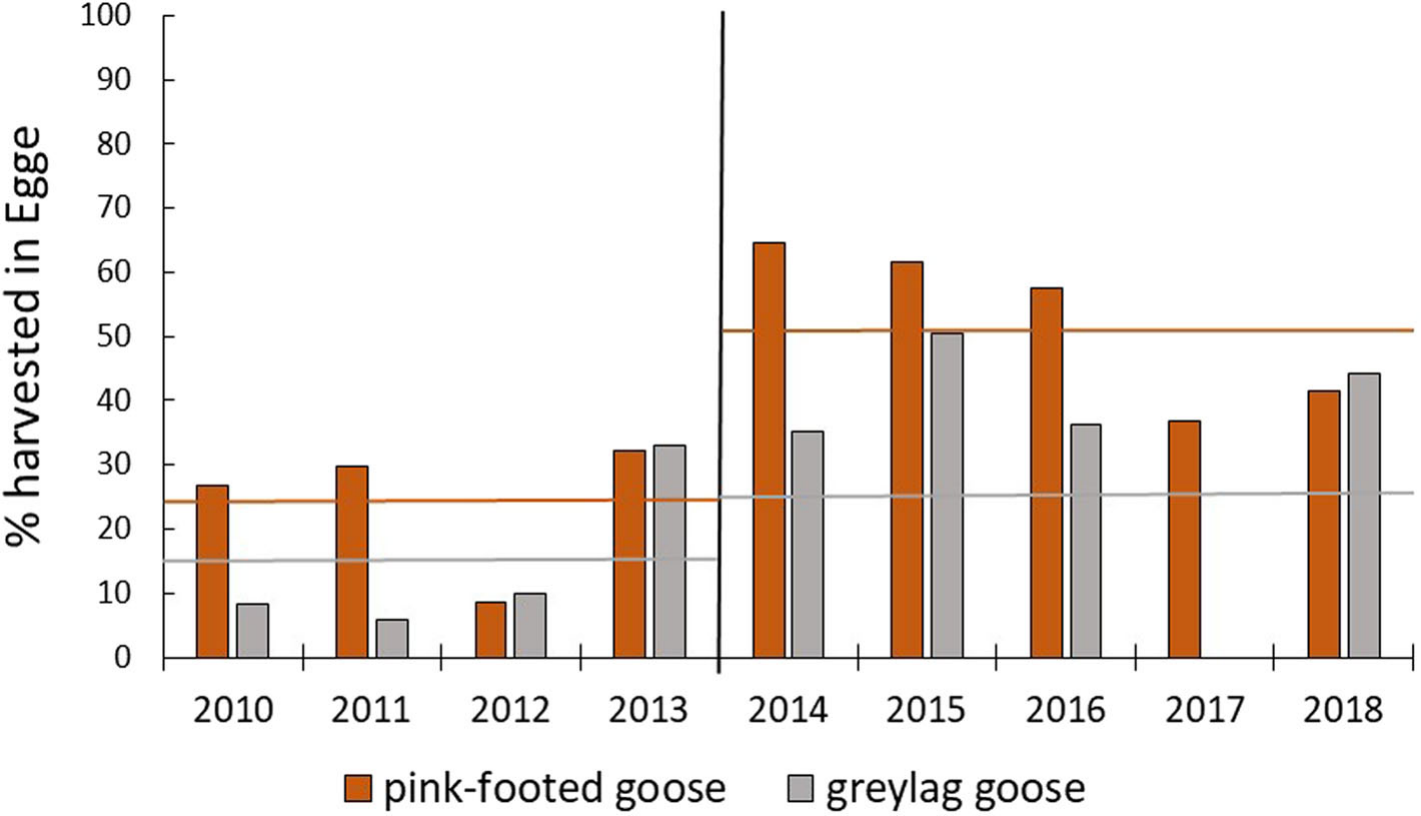
The annual percentage of pink-footed geese and greylag geese harvested in Egge, Trøndelag County, Norway, in 2010–2018, based on the total harvest of the species pink-footed geese in the whole municipality (Steinkjer 2019 data not yet available). In 2017 no greylag geese were harvested in Egge. From 2014 and onwards, only one hunting team was hunting, introducing hunting-free days between the hunts. The vertical line separates years of different hunting practices, and horizontal lines represent the averages within each period for the two goose species (see text for values)

Egge has been the only LOA in the municipality that actively organised goose hunting by the introduction of more hunting free days in the study period, and compared to the municipality harvest data the average percentage of harvested pink-footed geese is considerably less before 2014 than after (Fig. 4; before: on average 24% less, after: on average 52% less). Also the average percentage harvested greylag geese in Egge is considerably higher after the hunting was organised than before (Fig. 4; before: on average 14% less, after: on average 24% less).

**Fig. 5:**
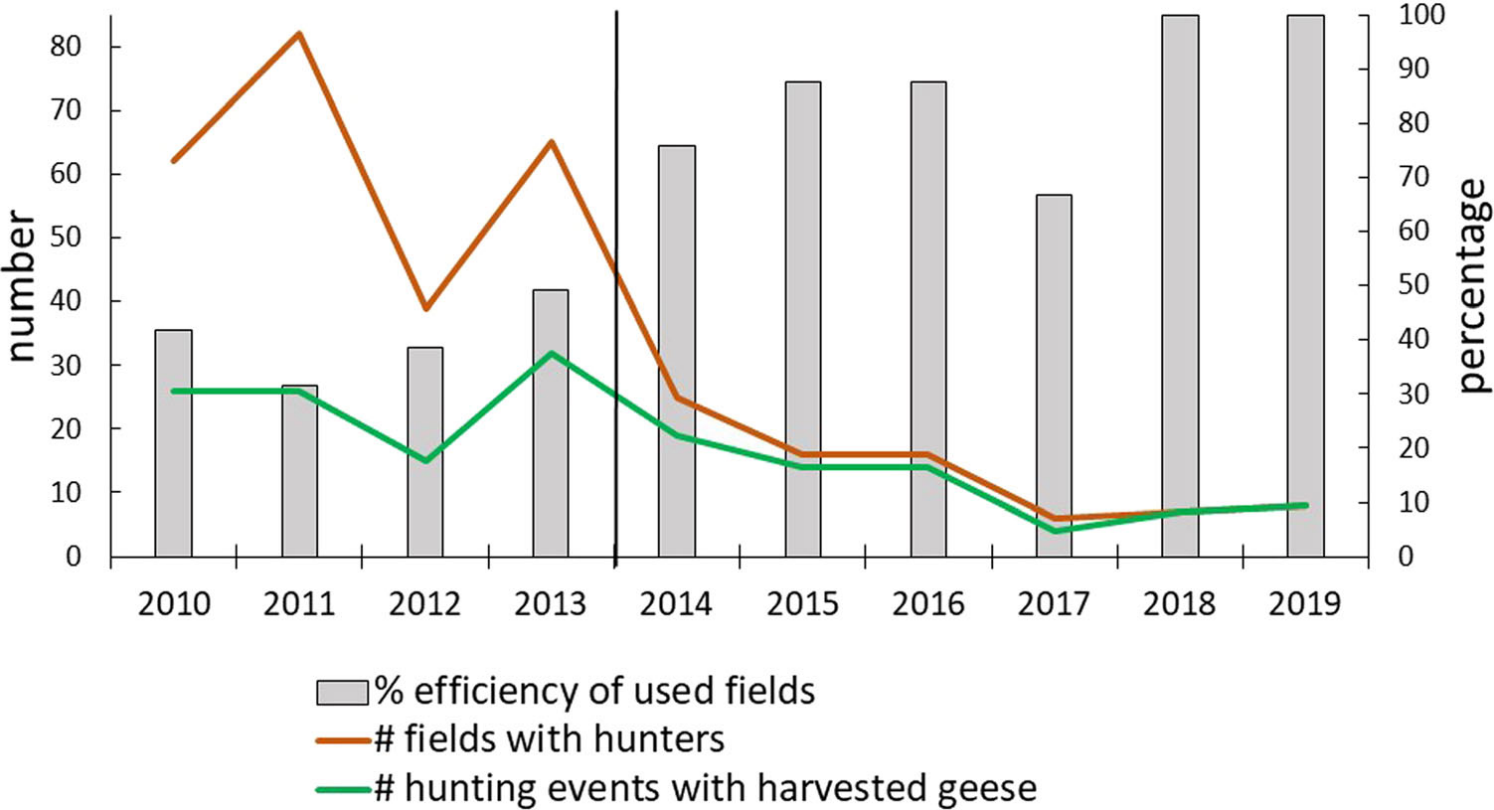
The number of fields in the hunting area occupied by hunters each year (2010–2019), and the number of fields where the hunters successfully shot one goose or more in a hunting area in Egge, Trøndelag County, Norway. Columns represent the efficiency of the fields used by hunters, in terms of successfully harvesting at least one goose per hunting event. The vertical line separates years of different hunting practices. From 2014 and onwards, only one hunting team was hunting, introducing hunting-free days between the hunts

The individual hunter’s efficiency in Egge was affected by the hunters’ positioning in the landscape (Fig. 5). The number of fields occupied by the hunters each year decreased over the study period (Table 2), and since the number of occupied fields with success, i.e. harvesting at least one goose each hunting event, also decreased (Table 2), the efficiency of used fields also increased (Table 2) and was 100% in 2018 and 2019 (Fig. 5).

**Fig. 6:**
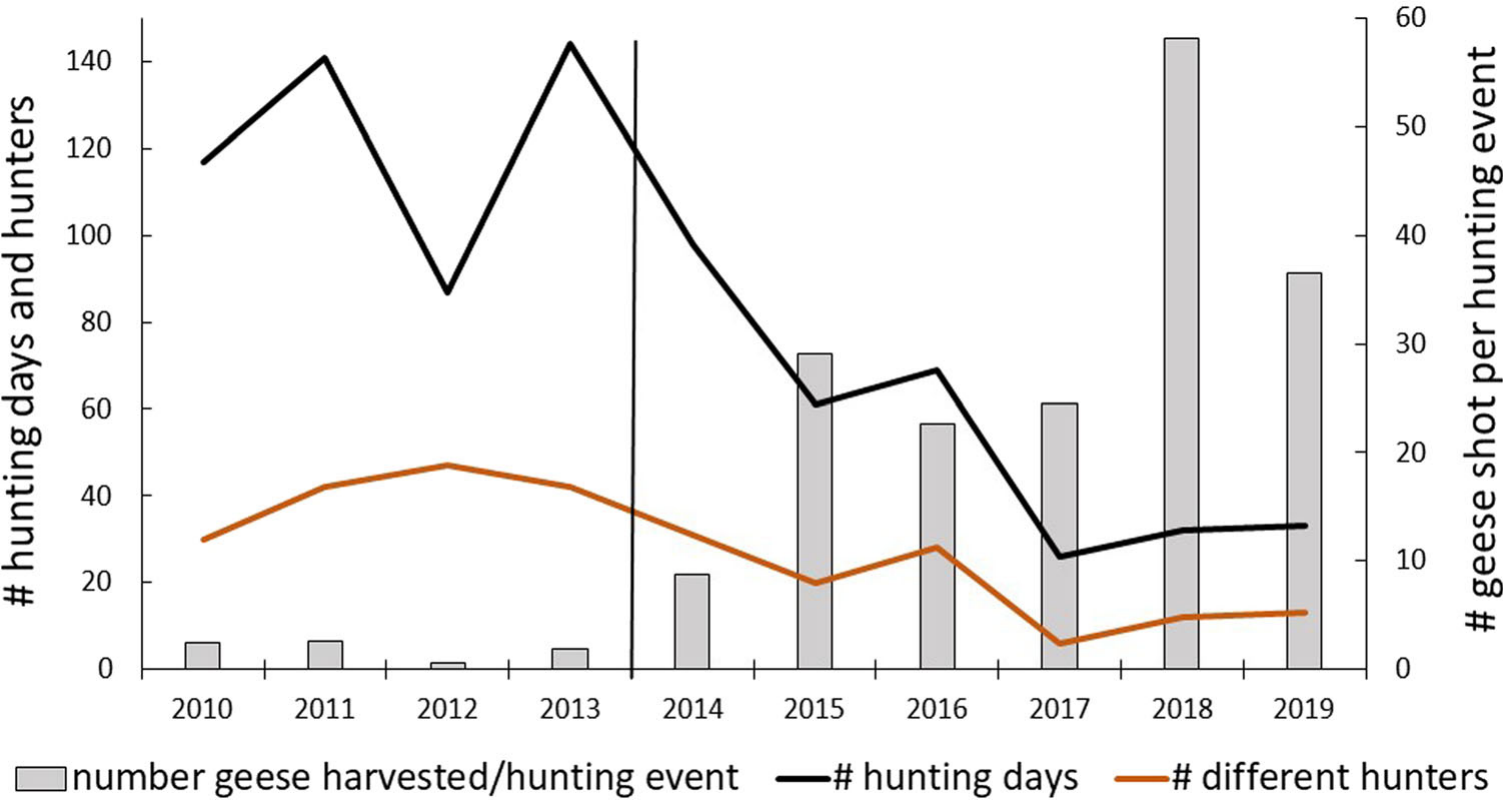
Number of days per year with goose hunting, number of different hunters and the number of geese harvested per hunting event in Egge, Trøndelag County, Norway. The vertical line separates years of different hunting practices. From 2014 and onwards, only one hunting team was hunting, introducing hunting-free days between the hunts

More efficient hunting is also illustrated by the number of geese harvested on each hunting day. Over the years the number of hunting days decreased (Fig. 6, Table 2), fewer hunters were hunting (Fig. 6, Table 2), and the bag size per hunting day significantly increased (Fig. 6, Table 2).

### Goose response to hunting

**Fig. 7:**
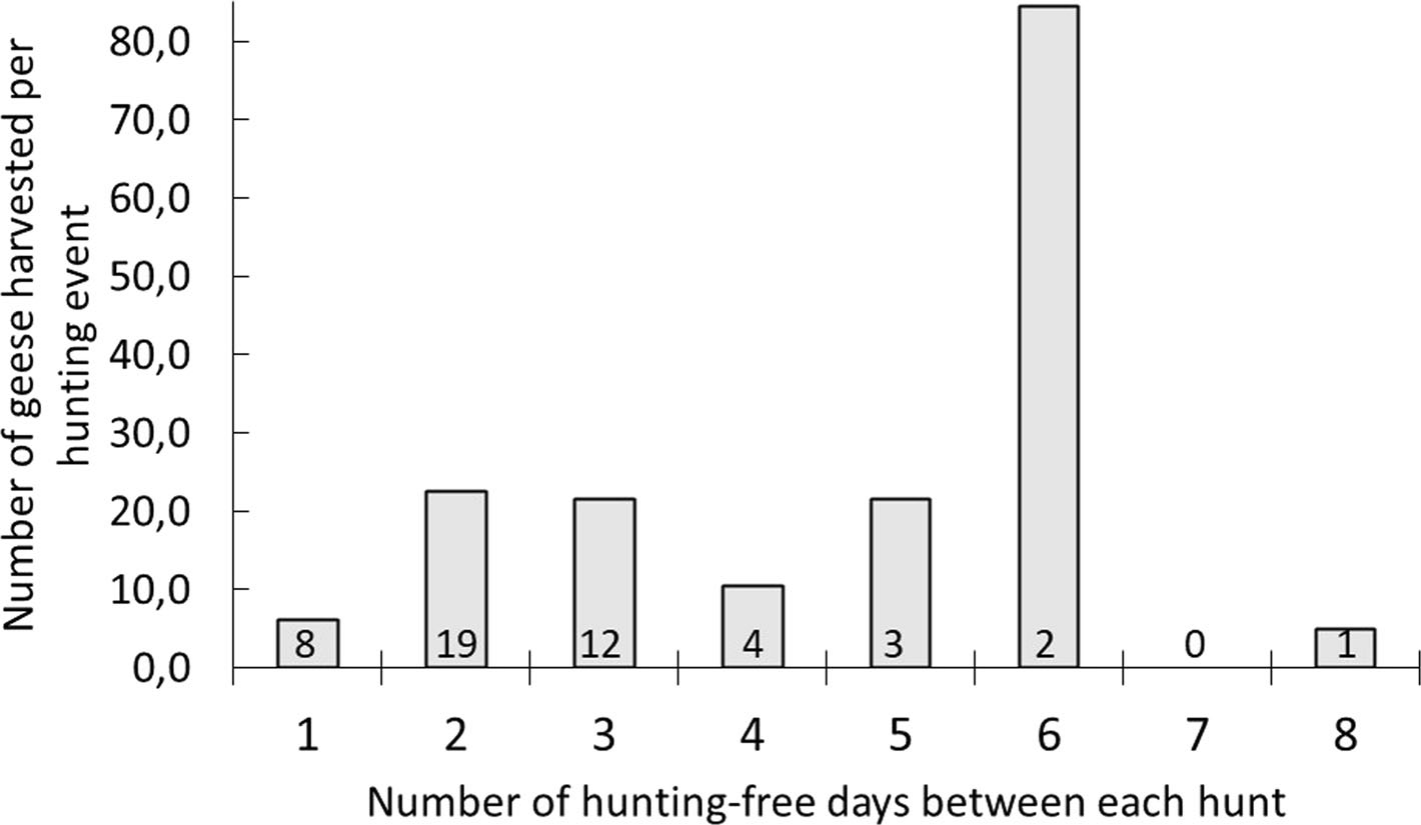
The number of harvested geese per hunting event in Egge, Trøndelag County, Norway, in relation to the number of hunting-free days between hunting events. Numbers within each column represent number of hunting events

The hunters’ behaviour and corresponding harvest rates can ultimately be explained by the behaviour of the geese and their responses to the hunting activities. By quantifying the distances between the fields used from one day to the next (pooled for 2014–2016), the goose flocks returned to the same field when there was no hunting in the area (within an average range of 119 ± 49 m, *n* = 65). Of these 65 days without hunting, the geese returned to the same spot in 56 of these occasions (86%, measured as 0 m away from field occupied the previous day). After days with goose hunting, on the other hand, the equivalent distances were significantly larger (on average 1 058 ± 129 m, *n* = 35, comparing distances between fields used by the geese on two consecutive days for days with and without hunting: *t* =  − 6.81, *p* < 0.001). After hunting days, geese were more than one kilometre away the following day at 21 of 35 occasions (60%). This behaviour affected the hunters’ bag sizes, and in Egge, the optimal number of hunting-free days between the hunts appeared to be two or three days and maybe also 5 days although this figure is based on fewer hunting days (Fig. 7). Moreover, in this case, one exception was when 133 geese were shot giving an average of 84.5 harvested geese based on two hunting days after six hunting-free days (Fig. 7).

## Discussion

When the management plan for the Svalbard-breeding pink-footed goose was endorsed (Madsen and Williams 2012), range states agreed on an adaptive decision making process following the principles of an adaptive harvest management programme (Nichols et al. 2007; Williams 2011). Hence, international-decided objectives must be realised at a local scale, and the present paper describes the adaptive process of local implementation. Increasing the harvest rate for geese was also a locally initiated effort due to increasing conflicts between geese and agriculture in the region, and the initiatives in Trøndelag worked in tandem with the international recommendations. Management objectives are presumably easier to accomplish when they, as in the present case, correspond with local interest (Hollow et al. 2014). Otherwise, processes are challenging when they contradict to local resource management (Redpath et al. 2013; Redpath et al. 2015). The successful increase in hunting bag in the LOA presented here is an example where both of these interests correspond.

The close co-operation and communication between local actors, managers and researchers are important factors for successful management (Riley et al. 2002; Chase et al. 2004; Elbroch et al. 2011; Callaghan et al. 2020; Henden et al. 2020). Stakeholder involvement is advantageous, since participation in management processes and transparent communication platforms strengthen the feelings of community and ownership (Nuno et al. 2014; Tuvendal and Elmberg 2015). For goose hunting in Trøndelag, being announced as an important management action also by local environmental authorities, several stakeholder groups have been represented in reference-groups of research projects focussing on geese in the region (Tombre et al. 2013a). In that way, research results, also with the contribution from stakeholders, were more easily communicated to end-users. This framework facilitates closer co-operation between stakeholder groups, local managers and researchers, and may, as in the present study, materialise in a common project focussing on relevant management actions.

As a wildlife management tool, studies have shown that hunters accept that hunting can be used as a measure for controlling goose populations (Dinges et al. 2014; Holmgaard et al. 2018). This is a fundamental premise for local actions if hunting is an important management issue. For the pink-footed goose population, where a population target is agreed, there may also be a need for less hunting at some point, and there must be a willingness among hunters to reduce their hunting activities in accordance with the current situation (Madsen et al. 2017). A survey among goose hunters in Trøndelag revealed the hunters’ interest to be involved in goose management as well as a willingness to reduce hunting effort. An important premise, however, was the wish to be informed about processes and being included in the communication loop (Holmgaard et al. 2018). Such a participatory policy provides several opportunities when implementing management actions (Decker et al. 2005; Newig and Fritsch 2009). Commonly, management decisions and strategies need local guidelines, input and experiences from those practically implementing the initiatives. Hence, the wish for goose hunters in Trøndelag to be more closely involved is both useful and reasonable. Being involved as a recreational hunter, has also demonstrated how voluntary agreements can regulate the local hunting activities in relation to protected areas and areas where hunting is allowed (Schou and Bregnballe 2006).

Information about optimal hunting regimes is also important for the farmland owners, as they are the actors, where hunting occurs on private land, setting the scene for hunting arrangements at their properties. Previous interviews and surveys among more than 300 farmland owners in Trøndelag revealed a motivated stakeholder group regarding hunting in general, and goose hunting in particular (Søreng et al. 2015). The majority meant that they also had a management responsibility and they were also positive to adapted goose hunting arrangements, a perspective that materialised in the arrangements and results from interviews described in the present study.

Although the case in Egge is an excellent example of how landowners can collaborate on a common objective with positive gains expected for all, there are several challenges keeping this together as an efficient unit. First of all, a significant time allocation is needed, not only to establish agreements and local hunting guidance, but also to keep all on board, motivated and with a common understanding of the aim of the arrangement. Unfortunate incidents, like too much shooting disturbance, may increase internal conflicts in the group and has also been a challenge in the present case. Keeping track of the harvest success throughout the season with a good information flow, including mandatory bag reports from hunters, will presumably increase the LOA’s engagement and motivation.

The present study does not only demonstrate how local wildlife management is initiated, and carried out, following objectives from international agreement. It also demonstrates, in this case increasing the autumn harvest of geese, how this specifically can be achieved. Although local adaptations are needed, results from Egge generate some rules-of thumbs that also match results elsewhere. Studies from Denmark revealed that locations with less shooting intensities and disturbance host more geese, and when there are shooting-free areas the number of geese increased significantly (Madsen 1998a, b, 2001). In a study on wildfowl, Bregnballe and Madsen (2004) also demonstrated that birds moved to neighbouring or more distant locations when there was shooting in one area. Disturbance from hunting may also change the goose migration movements and increase the flight distances between roosting sites and feeding areas (Madsen and Fox 1995; Béchet et al. 2003; Adams et al. 2016). Hence, less disturbance has positive effects on goose abundance, increasing the probabilities for hunting success (if hunting is allowed, i.e. not being in a protected area). In two LOAs in Trøndelag (other than Egge), increased harvest was also the result when, as in Egge, less hunting disturbance was practiced (Jensen et al. 2016a, b). Jointly, one recommendation, if the aim is to increase the harvest rate on autumn-staging geese, is therefore, to reduce hunting disturbance and temporally and spatially plan the activities in a way that always generate hunting-free areas for geese.

The number of harvested geese in Egge was significantly affected by disturbance, in terms of the number of hunting events, but the increasing abundance of geese over the study period also significantly influenced the number of geese harvested. The sudden increase from 2014 and onwards was not, however, significantly related to the overall increase in population size that nevertheless also increased gradually (Heldbjerg et al. 2019). This was analysed for pink-footed geese, that is the main goose species in the area. A shortcoming of our study is that goose registrations were gathered by non-professionals in the first years of the study period. We base our analyses on the extracted figures from an online platform where experienced ornithologists, known by name and known to be skilled bird observers, had entered their observations. Data were collected in the core period for goose staging and hunting, and we hence anticipate that these data are representative. Based on the data available in the present study, we suggest that a plausible explanation for the increasing hunting success in Egge is presumably a combination of (I) the effect of the within-season hunting activities providing several hunting-free days giving geese safe areas and opportunities to stay longer in the area, and (II) the increasing attractiveness of the area due to less disturbance. The latter may, therefore, cause an increase in the number of geese choosing the Egge area as a staging site in the autumn, a period where hunting disturbance is a significant factor when they migrate through Norway. The fact that harvested geese in Egge, as a fraction of the total harvest data for the whole municipality, also increased over the study period is a further indication that optimal goose hunting arrangement with hunting-free days will increase the harvest rate.

In correspondence with the present study, other studies have demonstrated how disturbance has a direct effect on whether geese return to the same field after being exposed to hunting. In a study by Jensen et al. (2016b), also conducted in Trøndelag, geese were registered more than one kilometre away from hunting fields the days after hunting, and the abundance and distribution of geese were directly influenced by the hunting activities. In Egge, when no hunting had taken place, the majority of geese came back to the same field the next day whereas on days after hunting, average distances between the hunting field and the goose flock were more than one kilometre also here. This behavioural response to hunting is presumably the main mechanism behind the reduced harvest after intensive hunting. Hunters may also, however, adapt to the situation not only by reducing the number of hunting events but also by the spatial distribution of hunting spots. In Egge, fewer hunters and hunting days, with targeted localisation of hunting fields increased the efficiency (harvesting at least one goose) of the fields in use. For example after four years of organised hunting, all fields used for hunting were successful the following years. The number of harvested geese per hunting event also increased by this practice, giving the highest number of geese after two or three hunting-free days although high success may occasionally be achieved after more days without hunting. Another recommendation for an optimal harvest arrangement is, therefore, to wait some days between each hunt and to use experienced goose hunters or outfitters with knowledge of local goose distribution and how to position themselves in the landscape. At Nesset, another LOA with organised goose hunting in Trøndelag (in Levanger municipality), three-days interval (or longer) is recommended (Jensen et al. 2016a) and at present only experienced goose hunters, following an organised hunting arrangement, are hunting in this area (Tombre et al. 2016).

## Conclusion

Adaptive management of wild goose populations depends on stakeholder collaboration and co-production of knowledge in an iterative learning-adaptation process. This study demonstrates how participation of motivated local end-users, in management processes aiming for common goals, can facilitate adaptive management and speed up its implementation. In the presented case, where goose hunting is applied as a population-regulating management tool, the active involvement of stakeholders in the planning, data collection and evaluation phases has been a significant contribution to the development of an optimal goose hunting arrangement, following the objectives agreed upon in an international management plan for pink-footed goose (Madsen and Williams 2012). The participatory framework for gaining and sharing knowledge has contributed to an increased sense of ownership of the outcomes, and to wider sharing of research findings and local experiences, to relevant audiences beyond management institutions and the scientific community. This study therefore demonstrates a successful stakeholder involvement in an adaptive process towards an optimal hunting arrangement for geese. These findings are also relevant for other cases of wildlife management, where management measures are implemented locally.
